# Random effects meta‐analysis: Coverage performance of 95*%* confidence and prediction intervals following REML estimation

**DOI:** 10.1002/sim.7140

**Published:** 2016-10-07

**Authors:** Christopher Partlett, Richard D. Riley

**Affiliations:** ^1^ National Perinatal Epidemiology UnitOxfordU.K.; ^2^ University of BirminghamBirminghamU.K.; ^3^ Research Institute for Primary Care and Health SciencesKeele UniversityKeeleU.K.

**Keywords:** random effects, meta‐analysis, coverage, REML, simulation

## Abstract

A random effects meta‐analysis combines the results of several independent studies to summarise the evidence about a particular measure of interest, such as a treatment effect. The approach allows for unexplained between‐study heterogeneity in the true treatment effect by incorporating random study effects about the overall mean. The variance of the mean effect estimate is conventionally calculated by assuming that the between study variance is known; however, it has been demonstrated that this approach may be inappropriate, especially when there are few studies. Alternative methods that aim to account for this uncertainty, such as Hartung–Knapp, Sidik–Jonkman and Kenward–Roger, have been proposed and shown to improve upon the conventional approach in some situations. In this paper, we use a simulation study to examine the performance of several of these methods in terms of the coverage of the 95*%* confidence and prediction intervals derived from a random effects meta‐analysis estimated using restricted maximum likelihood. We show that, in terms of the confidence intervals, the Hartung–Knapp correction performs well across a wide‐range of scenarios and outperforms other methods when heterogeneity was large and/or study sizes were similar. However, the coverage of the Hartung–Knapp method is slightly too low when the heterogeneity is low (*I* ^2^ < 30*%*) and the study sizes are quite varied. In terms of prediction intervals, the conventional approach is only valid when heterogeneity is large (*I* ^2^ > 30*%*) and study sizes are similar. In other situations, especially when heterogeneity is small and the study sizes are quite varied, the coverage is far too low and could not be consistently improved by either increasing the number of studies, altering the degrees of freedom or using variance inflation methods. Therefore, researchers should be cautious in deriving 95*%* prediction intervals following a frequentist random‐effects meta‐analysis until a more reliable solution is identified. © 2016 The Authors. *Statistics in Medicine* Published by John Wiley & Sons Ltd.

## 1 Introduction

A random effects meta‐analysis combines the results of several independent studies in order to summarise a particular measure of interest, such as a treatment effect. The approach allows for unexplained between‐study heterogeneity in the true treatment effect by incorporating random study effects about the overall mean 1, 2. Interest may be in estimating the overall mean (summary or pooled) effect and its confidence interval, quantifying the magnitude of heterogeneity itself or deriving predictive inferences about the treatment effect in a future setting, such as a 95*%* prediction interval or the probability the treatment will be effective 1.

There are numerous methods for constructing confidence intervals for a random effects meta‐analysis, and several articles have examined the performance of these methods. Cornell *et al.* 3 demonstrate a variety of methods in a classical example and show that the conventional approach can generate results with falsely high precision. Also, a number of simulation studies have also been carried out to compare the different methods. Previous simulation studies have focussed on comparing methods in terms of the coverage of the confidence intervals for the mean effect 4, 5, 6 or the significance level of the corresponding tests 7, 8, 9. These studies demonstrate that the conventional method, fit using either restricted maximum likelihood (REML) or DerSimonian–Laird 10, leads to too many significant results or overly precise confidence intervals.

However, when there is substantial heterogeneity between studies, prediction intervals are also useful for interpreting the results of a random effects meta‐analysis 1, 2. A prediction interval provides a description of the plausible range of effects if the treatment is applied in a new study or a new population similar to those included in the meta‐analysis. Indeed, when there is a large amount of variability between studies, the effect of the treatment in a new population could differ considerably from the mean effect, and the prediction interval should reflect this. Higgins *et al.* 1 and subsequently Riley *et al.* 2, suggest that the prediction interval is perhaps the most complete summary of a random effects meta‐analysis and propose an equation for its derivation (section 2.3).

In this paper, we build upon these previous studies by taking a more in depth look into the performance of several different methods for constructing confidence intervals for the mean effect and, in particular, prediction intervals for the effect in a new setting, following REML estimation of the random effects model in a frequentist framework. Firstly, we use a simulation study to examine the coverage performance of the confidence and prediction intervals for a number of different methods. Secondly, we provide an illustrative example to demonstrate how sensitive confidence and prediction intervals are to the methods used to construct them.

The outline of the paper is as follows. In section 2, we introduce the random effects meta‐analysis model and discuss some of the different methods for constructing confidence intervals for the average effect and prediction intervals for a new effect. In section 3, we provide details of our simulation study and present the results. In section 4, we illustrate the findings by applying the methods to an example data set. In section 5, we discuss the key findings and limitations and provide some recommendations regarding the use and construction of confidence and prediction intervals from meta‐analyses following REML estimation.

## 2 Random effects meta‐analysis

### 2.1 The random effects model

Suppose that there are *k* studies investigating a treatment effect, that is, the difference in outcome value or risk between a treatment group and a control group (for example, a mean difference, log odds ratio or a log hazard ratio). Further, suppose that each study provides the treatment effect estimate ${\hat{\theta }}_{i}$ and its variance $\sigma_{i}^{2}$, for *i* = 1,⋯,*k*. The random effects model assumes that, because of heterogeneity between studies, each study is potentially estimating a different true effect *θ*_*i*_. Therefore, the conventional parametric approach to random effects meta‐analysis, as outlined by Whitehead and Whitehead 11, is given by

(1) $$\begin{array}{cc} {\hat{\theta }}_{i} & ∼N\left(\theta_{i},\sigma_{i}^{2}\right),\\ \theta_{i} & ∼N(\theta ,\tau^{2}), \end{array}$$

where *θ* is the mean (also known as overall, summary or pooled) treatment effect, the $\sigma_{i}^{2}$ are assumed known and *τ* ^2^ is the between study variance of the treatment effects. There are numerous methods for fitting the random effects model (1); however, REML is often preferred, as (unlike ML estimation) it accounts for the simultaneous estimation of *τ* and *θ* and (unlike methods of moments 10 is based on normality of the random effects, which is convenient for making predictive inferences (see the discussion for more on this normality assumption).

### 2.2 Derivation of confidence intervals

Following estimation of model (1) using REML, the mean treatment effect estimate $\hat{\theta }$ is typically assumed to be normally distributed for large samples. Thus, a (1 − *α*)100*%* confidence interval for the mean effect *θ* is conventionally calculated by

(2) $$\hat{\theta }\pm Z_{\frac{\alpha }{2}}{\hat{\sigma }}_{\theta },$$

where $\hat{\theta }$ is the REML estimate of $\theta ,{\hat{\sigma }}_{\theta }$ is its standard error and $Z_{\frac{\alpha }{2}}$ is the upper $\frac{\alpha }{2}$ quantile of the standard normal distribution. However, ${\hat{\sigma }}_{\theta }$ does not account for uncertainty in the REML estimate of $\hat{\tau }$. Moreover, this approach also assumes that the within study variances are fixed and known, which is an untenable assumption when study sizes are small. As a result, Equation (2) may not provide an accurate reflection of the total uncertainty in the mean effect $\hat{\theta }$.

To address this, Hartung and Knapp 7 suggest using the adjusted confidence interval

(3) $$\hat{\theta }\pm t_{k-1;\frac{\alpha }{2}}\sqrt{{\mathrm{Var}}_{\text{HK}}},$$

where ${\mathrm{Var}}_{\text{HK}}=q{\hat{\sigma }}_{\theta }^{2},t_{k-1;\frac{\alpha }{2}}$ is the upper $\frac{\alpha }{2}$ quantile of the *t* distribution with *k* − 1 degrees of freedom and

$$q=\frac{1}{k-1}\overset{k}{\underset{i=1}{\sum }}w_{i}{\left(\hat{\theta }i-\hat{\theta }\right)}^{2},$$

where $w_{i}=\frac{1}{\sigma_{i}^{2}+{\hat{\tau }}^{2}}$ and ${\hat{\tau }}^{2}$ is the REML estimate of *τ* ^2^.

The Hartung–Knapp (HK) confidence interval in Equation (3) will usually be wider than the normal confidence interval (2) because it is based on the *t* distribution. However, it is possible, if *q* is sufficiently small, that the resulting interval will be shorter than the unadjusted interval. This obviously contradicts the idea that these intervals should be widened to account for additional uncertainty. With this in mind, Rover *et al.* 12 suggest a modification to the HK method which ensures that the resultant interval is not shorter than the conventional confidence interval in Equation (2). In particular, they suggest using

(4) $$\hat{\theta }\pm t_{k-1;\frac{\alpha }{2}}\sqrt{{\mathrm{Var}}_{\text{HK}}^{*}},$$

where ${\mathrm{Var}}_{\text{HK}}^{*}=q^{*}{\hat{\sigma }}_{\theta }^{2}$ and $q^{*}=\max\{1,q\}$.

Sidik and Jonkman 5 also independently suggested using (3) to calculate confidence intervals for *θ* and subsequently proposed an alternative modification to the HK method, which uses a more robust estimator of the variance. In particular, they suggest using

(5) $$\hat{\theta }\pm t_{k-1;\frac{\alpha }{2}}\sqrt{{\mathrm{Var}}_{\text{SJ}}},$$

where

$${\mathrm{Var}}_{\text{SJ}}=\frac{\overset{k}{\underset{i=1}{\sum }}w_{i}^{2}{\left({\hat{\theta }}_{i}-\hat{\theta }\right)}^{2}}{{\left(\overset{k}{\underset{i=1}{\sum }}w_{i}^{2}\right)}^{2}}.$$

The Sidik–Jonkman (SJ) confidence interval in Equation (5) will also usually be wider than the conventional confidence interval. However, Sidik and Jonkman 6 comment that Var_SJ_ is biased, especially for small *k* and so suggest an alternative confidence interval

(6) $$\hat{\theta }\pm t_{k-1;\frac{\alpha }{2}}\sqrt{{\mathrm{Var}}_{\text{SJ}}^{*}},$$

which uses the bias corrected estimator

$${\mathrm{Var}}_{\text{SJ}}^{*}=\frac{\overset{k}{\underset{i=1}{\sum }}w_{i}^{2}{\left(1-{ĥ}_{i}\right)}^{-1}{\left({\hat{\theta }}_{i}-\hat{\theta }\right)}^{2}}{{\left(\overset{k}{\underset{i=1}{\sum }}w_{i}^{2}\right)}^{2}},$$

where

$$h_{i}=\frac{2w_{i}}{\overset{k}{\underset{i=1}{\sum }}w_{i}}-\frac{\overset{k}{\underset{i=1}{\sum }}w_{i}^{2}\lambda_{i}^{2}}{\lambda_{i}^{2}{\left(\overset{k}{\underset{i=1}{\sum }}w_{i}\right)}^{2}}.$$

Another alternative is discussed by Kenward and Roger 9, which is implemented in the Stata package, ipdmetan 13. In particular, they suggest adjusting the variance of $\hat{\theta }$ using the expected information and appropriately modifying the degrees of freedom of the *t* distribution. For univariate random effects meta‐analysis, the expected information is given by

$$I_{\text{E}}=\frac{w_{2•}}{2}-\frac{w_{3•}}{w_{1•}}+\frac{1}{2}{\left(\frac{w_{2•}}{w_{1•}}\right)}^{2},$$

where

$$w_{j•}=\overset{k}{\underset{i=1}{\sum }}w_{i}^{j},\;j=1,2,3.$$

Then the adjusted variance is given by

$${\mathrm{Var}}_{\text{KR}}=\frac{1}{w_{1•}}+\frac{2\left(w_{3•}-\frac{w_{2•}}{w_{1•}}\right)}{w_{1•}^{2}I_{E}},$$

while the adjusted degrees of freedom are

$$\nu =\frac{2I_{E}}{{\left({\mathrm{Var}}_{\text{KR}}\cdot w_{2•}\right)}^{2}}.$$

Thus the Kenward–Roger (KR) confidence interval is given by

(7) $$\hat{\theta }\pm t_{\nu ;\frac{\alpha }{2}}\sqrt{{\mathrm{Var}}_{\text{KR}}}.$$

### 2.3 Derivation of prediction intervals

To summarise the potential treatment effect in a new setting, a (1 − *α*)100*%* prediction interval is conventionally calculated using the equation proposed by Higgins *et al.* 1,

(8) $$\hat{\theta }\pm t_{k-2;\frac{\alpha }{2}}\sqrt{{\hat{\tau }}^{2}+{\hat{\sigma }}_{\theta }^{2}},$$

where ${\hat{\tau }}^{2}$ is the REML estimate of the between study heterogeneity, while the variance of the mean effect ${\hat{\sigma }}_{\theta }^{2}$ is the conventional value obtained following REML estimation. However, in our simulations, we also consider modification of Equation (8) to replace ${\hat{\sigma }}_{\theta }^{2}$ with another measure, such as as ${\mathrm{Var}}_{\text{HK}}\left(\hat{\theta }\right)$ or ${\mathrm{Var}}_{\text{SJ}}\left(\hat{\theta }\right)$. In addition, although Kenward and Roger 9 do not discuss modifying Equation (7) to propose a prediction interval, a natural extension from their work is to replace Equation (8) with

(9) $$\hat{\theta }\pm t_{\nu -1;\frac{\alpha }{2}}\sqrt{{\hat{\tau }}^{2}+{\mathrm{Var}}_{\text{KR}}}.$$

## 3 Simulation study

### 3.1 Methods

We now perform a simulation study to examine the coverage performance of these aforementioned methods for deriving confidence and prediction intervals following REML estimation. The step by step guide to the simulation is summarised in the succeeding discussions.

Consider that a meta‐analysis of *k* trials is of interest, for summarising a treatment effect. For a set number of studies *k*, to simulate a meta‐analysis data set, we generate the *k* random effects *θ*_*i*_ from a normal distribution with mean *θ* and variance *τ* ^2^. The *k* within study variances ${\hat{\sigma }}_{i}^{2}$ are simulated from a chi‐square distribution centred on *σ* ^2^ and with *n* − 1 degrees of freedom. Here, *n* relates to the average within study sample size and controls the variability of the within study variance. We then generate the treatment effect estimates, ${\hat{\theta }}_{i}$, in each study from a normal distribution with mean *θ*_*i*_ and *σ* ^2^.

Next, we fit the random effects model (1) to the simulated data set, using REML, to obtain the estimates of the mean treatment effect *θ*, its variance ${\hat{\sigma }}_{\theta }^{2}$ and the between study variance *τ* ^2^ and use these to construct 95*%* confidence intervals for the mean effect using the conventional method (N) in Equation (2), the HK method in Equation (3), the modified HK method (HK2) in Equation (4), the SJ method in Equation (5), the bias corrected SJ method (SJ2) in Equation (6) and the KR method in Equation (7). We then determine whether the derived confidence intervals contain the true mean treatment effect *θ*. Similarly, we also construct 95*%* prediction intervals using Equation (8) for each of the HK and SJ methods and using Equation (9) for the KR method. We also simulate a new treatment effect from the true random effects distribution (second line in model (1)) and determine whether the prediction interval derived actually contains this new treatment effect.

The given process is then repeated 10 000 times for the chosen parameter values, to produce 10 000 meta‐analysis data sets and 10 000 confidence and prediction intervals for each method of interest. This allows us to calculate the coverage of the confidence and prediction intervals (that is, the proportion of times the true mean or the new treatment effect are found to lie in the derived confidence or prediction interval, respectively across all 10 000 replications).

Simulations were considered for a range of different scenarios that differed according to the parameter values chosen. The parameter values are summarised in Table 1. In particular, we varied the number of studies *k*, (either 3,5,7,10 or 100) and the relative degree of heterogeneity, controlled by $\nu =\frac{\tau^{2}}{\sigma^{2}}$. We consider a true mean treatment effect *θ* = 1 and an average within study variance *σ* ^2^ = 0.1 with the variability in the within study variance controlled by *n* = 30. The degree of heterogeneity is controlled using the ratio $\nu =\frac{\tau^{2}}{\sigma^{2}}$. In particular, we consider *τ* ^2^ = 1,0.1,0.05 and 0.01, which corresponds to *ν* = 10,1,0.5 and 0.1, respectively. However, we also report the average *I* ^2^ over each of the 10 000 meta‐analyses, to give the reader a feel for the relative magnitude of the between‐study variation relative to the total variation in the meta‐analysis for each scenario.

**Table 1:** Summary of the parameters used in the simulation study. In addition, we consider different sample size mixes around the average: one study 10 times larger, one study 10 times smaller and a mixture of large and small studies.

Parameter	Value(s)	Notes
*k*	3,5,7,10,100	Number of studies
*n*	30	Study sample size: controls the variability in the estimate of within study variance *σ* ^2^
*θ*	1	Average treatment effect
*σ* ^2^	0.1	True variance of effect estimates ${\hat{\theta }}_{i}$ for all studies. Estimates of *σ* ^2^ for each study are calculated using ${\hat{\sigma }}_{i}^{2}∼\frac{\sigma^{2}}{n-1}\chi_{n-1}^{2}=\frac{\sigma^{2}}{29}\chi_{29}^{2}$
*τ* ^2^	1,0.1,0.05,0.01	Expressed in terms of $\nu =\frac{\tau^{2}}{\sigma^{2}}=10,1,0.5,0.1$

In addition to the situation where all studies are of the same size (balanced: $\sigma_{i}^{2}=\sigma^{2}$), we also consider the impact of including one large study 10 times bigger than the others (a within study variance 10 times smaller: $\sigma_{i}^{2}=\sigma^{2}/10$) and one small study (with a within study variance 10 times larger: $\sigma_{i}^{2}=10\times \sigma^{2}$) and also the effect of a mixture of large and small studies. In these scenarios, we do not modulate *τ* ^2^ or *σ* ^2^ to account for modified sample size, and so, these modifications naturally impact the degree of heterogeneity in these settings. It is also important to note that when the study sizes are unbalanced, one must be careful when interpreting *ν* and *I* ^2^ as both these measures of heterogeneity rely on the idea of a ‘typical’ within study variance.

With 10 000 studies, the Monte Carlo error is approximately 0.4, and so, if the coverage is indeed 0.95, coverage outside the range of [94.6,95.4] is unexpected.

### 3.2 Results

**Figure 1:**
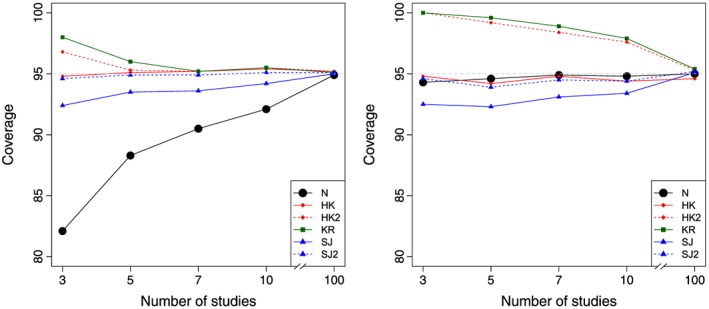
Confidence interval coverage based on a random effects meta‐analysis fit using REML for balanced studies with a large degree of heterogeneity *ν* = 10 (left) and low degree of heterogeneity *ν* = 0.1 (right), for each of the different methods. Methods: N, conventional method; HK, Hartung–Knapp; HK2, modified Hartung–Knapp; KR, Kenward–Roger; REML, restricted maximum likelihood; SJ, Siddik–Jonkman; SJ2, modified Sidik–Jonkman.

**Figure 2:**
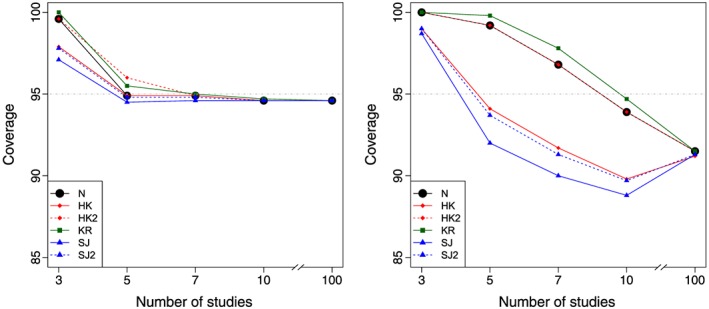
Prediction interval coverage based on a random effects meta‐analysis fit using REML for balanced studies with a large degree of heterogeneity *ν* = 10 (left) and low degree of heterogeneity *ν* = 0.1 (right), for each of the different methods. Methods: N, conventional method; HK, Hartung–Knapp; HK2, modified Hartung–Knapp; KR, Kenward–Roger; REML, restricted maximum likelihood; SJ, Siddik–Jonkman; SJ2, modified Sidik–Jonkman.

Tables 2, 3, 4, 5 display the coverage of the 95*%* confidence and prediction intervals for each of the approaches, for each of a variety of scenarios defined by different *k* and *ν*. The tables also report the average *I* ^2^ over each of the 10 000 simulations, to give the reader a feel for the relative magnitude of the between‐study variation relative to the total variation in the meta‐analysis for each scenario. Figures 1 and 2 graphically display the confidence and prediction interval coverage, respectively, for each of the methods, based on balanced studies with a large (*ν* = 10) and small (*ν* = 0.1) degree of heterogeneity.

**Table 2:** The coverage of the 95*%* confidence interval (CI) and prediction interval (PI) obtained by fitting the model (1) using restricted maximum likelihood (N) and the coverage of the adjusted CI and PI using a variety of methods. The results are based on a random effects meta‐analysis with *k* studies and *ν* = 10. The table also includes the average *I* ^2^ across each of the 10 000 simulations.

Method			Balanced studies						One small study				
			*k* = 3	*k* = 5	*k* = 7	*k* = 10	*k* = 100		*k* = 3	*k* = 5	*k* = 7	*k* = 10	*k* = 100
CI	N		82.1	88.3	90.5	92.1	94.9		79.8	86.8	89.5	92.2	94.6
	HK		94.8	95.1	95.2	95.4	95.2		92.1	94.0	94.5	95.3	94.9
	HK2		96.8	95.9	95.2	95.5	95.2		96.0	94.7	94.9	95.6	94.9
	KR		98.0	96.0	95.2	95.5	95.1		97.0	96.2	95.2	95.6	94.9
	SJ		92.4	93.2	93.6	94.2	95.0		87.8	91.9	92.9	94.5	94.8
	SJ2		94.6	94.9	94.9	95.1	95.1		85.4	88.0	90.1	93.0	94.8
PI	N		99.6	94.9	94.9	94.6	94.6		99.4	94.0	94.3	94.8	94.8
	HK		97.9	94.9	94.9	94.6	94.6		96.8	93.8	94.3	94.8	94.8
	HK2		99.6	96.0	94.9	94.6	94.6		99.4	94.1	94.4	94.8	94.8
	KR		100.0	95.5	95.0	94.7	94.6		99.7	97.0	95.0	95.0	94.8
	SJ		97.1	94.5	94.6	94.6	94.6		94.0	93.2	94.1	94.8	94.8
	SJ2		97.8	94.8	94.8	94.6	94.6		94.9	93.1	94.0	94.7	94.8
*I* ^2^(*%*)			74.8	83.9	87.1	88.9	91.3		65.2	80.4	85.3	88.0	91.3
Method			One large study						Mixture of study sizes				
			*k* = 3	*k* = 5	*k* = 7	*k* = 10	*k* = 100		*k* = 3	*k* = 5	*k* = 7	*k* = 10	*k* = 100
CI	N		80.9	87.2	90.2	92.2	94.7		73.6	84.0	88.2	90.7	95.1
	HK		93.6	94.2	94.8	95.3	95.0		85.3	90.8	93.1	93.4	94.9
	HK2		94.7	94.5	94.9	95.4	95.0		88.3	92.1	94.0	94.3	95.4
	KR		98.7	96.0	95.2	95.3	95.0		95.2	95.8	96.0	95.0	95.4
	SJ		90.6	92.3	93.3	94.1	94.9		80.0	88.7	91.5	92.6	95.2
	SJ2		89.9	91.1	92.7	93.8	94.9		66.2	67.8	69.7	71.1	80.5
PI	N		97.9	95.2	94.8	94.7	94.7		94.1	92.1	93.6	94.7	96.4
	HK		96.9	95.1	94.8	94.7	94.7		91.6	92.0	93.7	94.6	96.4
	HK2		97.9	95.2	94.8	94.7	94.7		94.4	92.3	93.8	94.7	96.4
	KR		99.9	97.9	95.5	94.8	94.7		97.9	98.6	97.5	96.0	96.4
	SJ		95.3	94.9	94.5	94.6	94.7		86.6	91.5	93.4	94.5	96.4
	SJ2		95.3	94.8	94.6	94.6	94.7		86.5	90.5	92.5	93.9	96.4
*I* ^2^(*%*)			81.7	88.4	90.5	91.5	91.9		71.1	90.4	95.0	96.7	98.2

**Table 3:** The coverage of the 95*%* confidence interval (CI) and prediction interval (PI) obtained by fitting the model (1) using restricted maximum likelihood (N) and the coverage of the adjusted CI and PI using a variety of methods. The results are based on a random effects meta‐analysis with *k* studies and *ν* = 1. The table also includes the average *I* ^2^ across each of the 10 000 simulations.

Method			Balanced studies						One small study				
			*k* = 3	*k* = 5	*k* = 7	*k* = 10	*k* = 100		*k* = 3	*k* = 5	*k* = 7	*k* = 10	*k* = 100
CI	N		89.6	91.0	92.2	92.9	94.7		88.4	90.9	91.9	92.6	94.8
	HK		94.6	94.8	94.9	94.7	94.8		93.4	94.1	94.7	94.9	94.9
	HK2		99.8	97.7	96.7	95.9	95.0		99.7	97.6	96.6	95.9	95.1
	KR		100	98.4	97.2	96.1	95.0		99.9	99.3	97.5	96.3	95.1
	SJ		92.1	92.8	93.4	93.9	94.8		87.5	91.5	92.9	93.7	95.0
	SJ2		94.3	94.6	94.7	94.7	94.9		89.4	92.6	93.7	94.3	95.0
PI	N		100	92.8	89.0	88.3	94.9		100	93.4	89.5	89.1	95.3
	HK		98.6	89.6	87.5	87.5	94.9		98.4	89.5	87.8	88.5	95.3
	HK2		100	92.9	89.1	88.3	94.9		100	93.5	89.8	89.3	95.3
	KR		100	96.0	90.5	88.7	94.9		100	98.9	92.5	90.3	95.3
	SJ		97.8	87.9	86.4	86.9	94.9		96.0	87.3	86.5	88.0	95.3
	SJ2		98.5	89.3	87.2	87.4	94.9		96.8	88.4	87.2	88.3	95.3
*I* ^2^(*%*)			33.2	38.7	40.9	43.1	52.5		33.7	39.3	42.0	44.6	52.8
Method			One large study						Mixture of study sizes				
			*k* = 3	*k* = 5	*k* = 7	*k* = 10	*k* = 100		*k* = 3	*k* = 5	*k* = 7	*k* = 10	*k* = 100
CI	N		78.9	85.0	88.2	89.9	94.6		77.1	84.0	87.8	90.8	94.4
	HK		89.1	90.7	92.2	92.9	94.7		85.8	89.9	92.0	93.9	95.5
	HK2		96.5	93.6	94.1	93.8	94.9		94.0	92.7	93.6	95.1	95.6
	KR		100	100	99.5	98.4	94.9		97.5	97.4	97.4	96.9	95.0
	SJ		79.1	85.0	88.5	90.6	94.8		73.9	85.7	89.6	92.1	94.6
	SJ2		78.5	82.9	86.6	89.4	94.7		65.5	66.6	69.3	72.0	86.9
PI	N		100	87.2	87.0	88.1	94.5		99.6	89.8	91.6	94.1	98.8
	HK		95.8	85.5	86.4	87.8	94.5		94.3	89.2	91.6	94.1	98.9
	HK2		100	87.5	87.3	88.3	94.5		99.7	90.3	92.0	94.3	98.9
	KR		100	100	99.8	98.3	94.5		99.7	99.9	98.4	97.0	98.9
	SJ		88.3	81.9	84.3	86.7	94.5		81.2	86.7	90.8	93.8	98.8
	SJ2		88.3	81.3	83.8	86.6	94.5		81.3	85.9	90.0	93.0	98.8
*I* ^2^(*%*)			41.6	47.3	49.8	51.6	54.0		48.8	66.7	74.1	79.6	87.9

**Table 4:** The coverage of the 95*%* confidence interval (CI) and prediction interval (PI) obtained by fitting the model (1) using restricted maximum likelihood (N) and the coverage of the adjusted CI and PI using a variety of methods. The results are based on a random effects meta‐analysis with *k* studies and *ν* = 0.5. The table also includes the average *I* ^2^ across each of the 10 000 simulations.

Method			Balanced studies						One small study				
			*k* = 3	*k* = 5	*k* = 7	*k* = 10	*k* = 100		*k* = 3	*k* = 5	*k* = 7	*k* = 10	*k* = 100
CI	N		92.3	92.4	92.7	92.7	95.1		91.6	92.2	93.0	93.0	94.4
	HK		94.7	94.7	94.1	94.3	95.0		94.5	94.1	94.8	94.5	94.4
	HK2		99.9	98.6	97.0	96.1	95.4		99.9	98.5	97.6	96.3	94.7
	KR		100	99.2	97.7	96.4	95.4		100	99.6	98.6	96.9	94.7
	SJ		92.1	92.4	92.5	93.2	95.3		88.7	91.2	92.9	93.2	94.6
	SJ2		94.3	94.4	93.7	94.2	95.3		90.7	92.5	93.9	93.9	94.6
PI	N		100	95.0	89.6	87.2	94.5		100	95.6	91.4	88.5	94.8
	HK		98.6	89.7	86.1	85.5	94.5		98.9	90.3	88.7	86.8	94.8
	HK2		100	95.1	89.7	87.3	94.5		100	95.8	91.8	88.6	94.8
	KR		100	97.5	91.6	88.1	94.5		100	99.6	94.8	90.1	94.8
	SJ		97.8	87.6	84.6	84.6	94.5		97.0	87.2	86.7	85.7	94.8
	SJ2		98.6	89.4	85.9	85.3	94.5		97.5	88.8	87.8	86.4	94.8
*I* ^2^(*%*)			25.7	27.7	28.6	30.2	36.4		29.7	31.5	32.5	33.4	37.3
Method			One large study						Mixture of study sizes				
			*k* = 3	*k* = 5	*k* = 7	*k* = 10	*k* = 100		*k* = 3	*k* = 5	*k* = 7	*k* = 10	*k* = 100
CI	N		81.8	86.2	88.6	91.0	95.2		80.5	85.8	88.7	90.5	94.7
	HK		89.7	90.2	91.3	92.8	95.2		88.2	90.6	92.8	93.8	96.3
	HK2		98.3	95.0	94.1	94.6	95.6		96.9	94.1	94.6	95.1	96.4
	KR		100	100	99.9	99.5	95.6		98.7	98.7	98.3	97.0	95.2
	SJ		77.7	82.7	86.3	89.8	95.4		74.1	85.1	89.6	91.8	94.9
	SJ2		77.1	80.5	84.1	88.3	95.4		68.3	70.5	73.2	75.9	89.8
PI	N		100.0	88.6	85.1	84.8	94.6		100	89.9	90.3	92.9	99.3
	HK		96.9	84.8	83.4	83.9	94.6		95.9	88.6	90.5	93.0	99.3
	HK2		100	89.0	85.4	85.1	94.6		100	90.7	91.1	93.2	99.3
	KR		100	100	100	99.7	94.7		100	100	97.9	95.9	99.3
	SJ		89.7	78.3	79.5	81.7	94.6		83.0	84.8	88.9	92.5	99.3
	SJ2		89.6	77.8	79.0	81.4	94.6		83.0	84.2	88.1	91.8	99.3
*I* ^2^(*%*)			31.3	34.8	36.1	37.0	37.9		43.0	56.9	64.3	69.7	81.9

**Table 5:** The coverage of the 95*%* confidence interval (CI) and prediction interval (PI) obtained by fitting the model (1) using restricted maximum likelihood (N) and the coverage of the adjusted CI and PI using a variety of methods. The results are based on a random effects meta‐analysis with *k* studies and *ν* = 0.1. The table also includes the average *I* ^2^ across each of the 10 000 simulations.

Method			Balanced studies						One small study				
			*k* = 3	*k* = 5	*k* = 7	*k* = 10	*k* = 1004		*k* = 3	*k* = 5	*k* = 7	*k* = 10	*k* = 100
CI	N		94.3	94.6	94.9	94.8	95.0		93.9	94.6	94.9	94.4	94.7
	HK		94.8	94.2	94.8	94.4	94.6		94.8	94.8	94.9	94.6	94.2
	HK2		100	99.2	98.4	97.6	95.3		99.8	99.1	98.2	97.4	94.9
	KR		100	99.6	98.9	97.9	95.4		100	99.8	99.2	98.0	94.9
	SJ		92.5	92.3	93.1	93.4	95.1		88.9	91.7	93.0	93.1	94.7
	SJ2		94.6	93.9	94.5	94.4	95.2		91.0	93.1	93.8	93.8	94.7
PI	N		100	99.2	96.8	93.9	91.5		100	99.1	97.0	94.4	92.3
	HK		99.0	94.1	91.7	89.8	91.2		99.2	94.9	92.8	90.9	92.0
	HK2		100	99.2	96.8	93.9	91.5		100	99.2	97.1	94.6	92.3
	KR		100	99.8	97.8	94.7	91.5		100	100	98.9	95.7	92.3
	SJ		98.7	92.0	90.0	88.8	91.3		97.8	92.3	90.8	89.5	92.1
	SJ2		99.0	93.7	91.3	89.7	91.3		98.2	93.5	91.9	90.2	92.1
*I* ^2^(*%*)			18.0	17.7	17.6	17.4	14.7		25.1	24.4	23.0	22.0	16.3
Method			One large study						Mixture of study sizes				
			*k* = 3	*k* = 5	*k* = 7	*k* = 10	*k* = 100		*k* = 3	*k* = 5	*k* = 7	*k* = 10	*k* = 100
CI	N		90.6	91.8	92.1	92.3	94.7		90.8	91.8	92.2	93.4	94.9
	HK		93.1	92.1	92.2	92.5	94.3		93.5	94.1	94.7	95.8	97.2
	HK2		99.9	98.0	96.9	96.0	94.9		99.9	98.3	97.4	97.3	97.3
	KR		100	100	100	99.9	95.4		100	100	99.6	98.6	95.4
	SJ		80.5	82.9	84.3	87.4	94.6		76.7	85.8	89.2	92.2	94.9
	SJ2		80.4	81.0	82.6	86.3	94.5		74.9	78.6	81.9	85.1	92.3
PI	N		100	96.1	91.9	89.6	90.5		100	95.0	91.4	90.7	99.3
	HK		98.6	89.5	86.7	86.0	90.3		98.7	91.4	89.8	90.8	99.3
	HK2		100	96.3	92.3	89.9	90.5		100	95.9	92.3	91.8	99.3
	KR		100	100	100	100	90.7		100	100	99.1	95.5	99.3
	SJ		95.1	79.6	78.8	80.3	90.3		88.2	82.6	84.6	87.6	99.3
	SJ2		95.0	78.8	77.9	79.6	90.3		87.7	82.5	84.1	87.5	99.3
*I* ^2^(*%*)			20.0	19.9	19.9	19.0	15.2		33.6	39.8	44.1	48.7	68.9

### 3.3 Performance of confidence intervals

For relatively large heterogeneity (ν⩾1), the conventional confidence intervals (Equation (2) are consistently too short when there are only a small number of studies. For example, in Table 2(*ν* = 10), the conventional confidence intervals have coverage of 82*%* and 88*%* for *k* = 3(*I* ^2^ = 75*%*) and *k* = 5(*I* ^2^ = 84*%*) balanced studies, respectively. These results agree with previous simulations studies 5, 6, 7. The coverage of the conventional confidence intervals improves as *k* increases, but at least 10 studies are required to obtain coverage, which is reasonably close to 95*%*. For example, in Table 3(*ν* = 1), the conventional confidence intervals have coverage of 93*%* and 95*%* for *k* = 10(*I* ^2^ = 43*%*) and *k* = 100(*I* ^2^ = 52*%*) balanced studies, respectively.

All of the modified methods (Equations (3)–(7) perform reasonably well in terms of confidence interval coverage provided k⩾5. For example, in Table 2(*ν* = 10), the coverage probabilities of the HK method is 95*%*, while the HK2 confidence interval is slightly wider on average with coverage 96*%*. Similarly, the KR and SJ methods are 96*%* and 93*%* respectively for *k* = 5(*I* ^2^ = 84*%*) balanced studies, compared with 88*%* for the conventionally calculated confidence intervals. In general, the KR method seems to produce confidence intervals that are slightly too wide on average, while the SJ confidence intervals are slightly too short on average; however, as *k* increases, both these converge on the expected coverage of 95*%*.

When there is very little heterogeneity, the conventional confidence intervals perform much better. For example, in Table 5(*ν* = 0.1), the conventional confidence interval has reasonably good coverage in almost all cases and is comparable to the HK method. In this case, the KR and HK2 methods generate confidence intervals that are slightly too wide when there are a small number of studies. For example, for *k* = 10(*I* ^2^ = 17*%*) balanced studies, the KR and HK2 methods both have coverage of 98*%*.

Of all the modified methods, the HK method is frequently the best in terms of confidence interval coverage; however, when the heterogeneity is small and the study sizes are mixed, the HK method produces confidence intervals that are too wide. For example, in Table 5(*ν* = 0.1), the coverage probabilities of the HK and HK2 methods are both 97*%* for *k* = 100(*I* ^2^ = 69*%*) studies with a mixture of sizes, compared with 95*%* for the conventionally calculated confidence intervals.

### 3.4 Performance of prediction intervals

Prediction interval coverage is reasonably good for all methods *provided* that the relative degree of heterogeneity is reasonably large (*ν* > 1;*I* ^2^ > 30*%* approximately for balanced studies) *and* there are at least *k* = 5 studies. For example, in Table 2(*ν* = 10) for *k* = 3(*I* ^2^ = 75*%*) balanced studies, the coverage is too large, but for $k⩾5\;(I^{2}⩾84\%)$, the prediction interval coverage is close to 95*%* for all methods.

However, the coverage performance of the prediction intervals is poor in situations where the relative degree of heterogeneity is small or moderate. For example, in Table 3 where *ν* = 1, there is departure from the expected coverage of 95*%* for all methods when *k*⩽10, including the conventional method (Equation (8), with coverage often considerably less than 95*%*. Similarly, for *ν* < 1(*I* ^2^ < 30*%* approximately for balanced studies), there are even more serious departures from the expected coverage of 95*%*. For example, in Table 4, the conventional method has coverage of 87*%* for *k* = 10(*I* ^2^ = 30*%*) balanced studies. Also, in Table 5, the prediction interval coverage is 91*%* for all methods even when there are *k* = 100(*I* ^2^ = 15*%*) balanced studies.

Coverage of prediction intervals is also poor when there are a mixture of large and small studies. For example, for *ν* = 0.1 and *k* = 100(*I* ^2^ = 69*%*), the prediction intervals are generally too wide, with coverage of 99*%* for all methods. Changing the degrees of freedom used in Equation (8), for example, to *k* − 3 rather than *k* − 2, did not consistently improve coverage performance to the required level (results not shown).

## 4 Example

We consider a meta‐analysis of seven randomised trials looking into the effect of anti‐hypertensive treatment versus control on reducing diastolic blood pressure (DBP) in patients with hypertension, as detailed elsewhere 14. The treatment effect of interest is the difference in the final mean DBP value between the treatment and control groups, after adjusting for baseline DBP. Treatment effect estimates and their variances were derived in each study, using analysis of covariance as detailed by Riley *et al.* 15 and a random‐effects meta‐analysis used to synthesise the results using REML. Heterogeneity is expected given the diversity of included populations. Crucially, there is also a mixture of study sizes, with the largest having 6991 participants and the smallest having only 172.

Additionally, for illustrative purposes, we also took the mean difference between treatment groups at baseline in each study and then performed a random effects meta‐analysis for this measure 15. This was done to consider a situation where homogeneity is expected, such that the relative amount of between‐study variance should be very small.

In each meta‐analysis, we construct confidence and prediction intervals using each of the methods outlined in the previous section, and the findings are now described.

### 4.1 Pre‐treatment meta‐analysis

**Figure 3:**
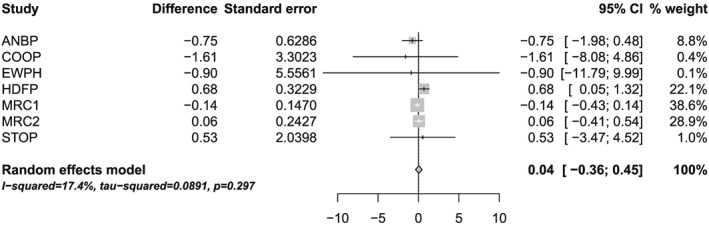
Forest plot for the pre‐treatment difference in diastolic blood pressure between the treatment and control groups based on the random effects meta‐analysis model (1) fit using restricted maximum likelihood and confidence interval for the mean treatment effect calculated using the conventional method.

**Figure 4:**
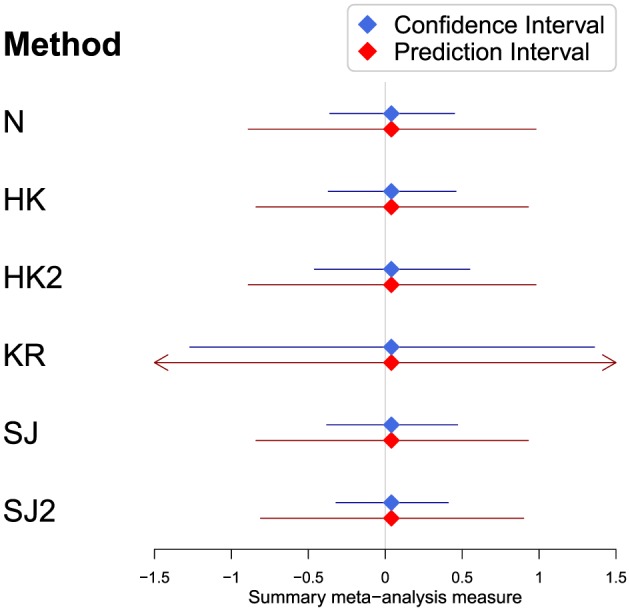
The summary results for the pre‐treatment difference in diastolic blood pressure between the treatment and control groups based on a random effects meta‐analysis fit using restricted maximum likelihood with confidence and prediction intervals constructed using a variety of methods.

The forest plot in Figure 3 shows the results of the REML random effects meta‐analysis (model (1) on the pre‐treatment difference in DBP between the treatment and control groups. As expected, prior to treatment, there is a relatively low degree of heterogeneity between studies with *I* ^2^ = 17.4*%*[0*%*;61.3*%*] and $\hat{\tau }=0.09$. Table 6 and Figure 4 summarise the confidence and prediction intervals constructed using each of the methods. Unsurprisingly, the summary mean difference is very small indicating that the two randomised groups are well balanced in terms of baseline DBP. All the derived 95*%* confidence intervals are reasonably similar, with the exception of the KR method, which is much wider than the others. As identified in the simulation study, the KR method generally produces slightly wider confidence intervals in this setting, namely, when there are a mixture of study sizes and a relatively low degree of heterogeneity. Almost all of the adjustment methods successfully generate wider confidence intervals than the conventional method, but the SJ2 confidence interval actually shrinks compared with the conventional method. Again, this is identified in the simulation study as the coverage of the SJ2 method is too small in this setting.

**Table 6:** The pre‐treatment difference in diastolic blood pressure between the treatment and control groups $\left({\hat{\theta }}_{0}\right)$ along with the 95*%* confidence intervals (CI) and standard error (s.e.) for each study. The summary results $\hat{\theta }$ are based on a random effects meta‐analysis fit using restricted maximum likelihood with confidence and prediction intervals constructed using a variety of methods. Methods: N, conventional method; HK, Hartung–Knapp; HK2, modified Hartung–Knapp; KR, Kenward–Roger; SJ, Siddik–Jonkman; SJ2, modified Sidik–Jonkman.

Individual study results				
Trial	${\hat{\theta }}_{0}$	95*%* CI	s.e.	*n*
ANBP	− 0.75	[ − 1.98; 0.48 ]	0.63	1530
COOP	− 1.61	[ − 8.08; 4.86 ]	3.30	349
EWPH	− 0.90	[ − 11.79; 9.99 ]	5.56	172
HDFP	0.68	[ 0.05; 1.32 ]	0.32	4797
MRC1	− 0.14	[ − 0.43; 0.14 ]	0.15	6991
MRC2	0.06	[ − 0.41; 0.54 ]	0.24	2651
STOP	0.53	[ − 3.47; 4.52 ]	2.04	268
Summary meta‐analysis results				
Method	$\hat{\theta }$	95*%* CI	95*%* PI	
N	0.04	[ − 0.36; 0.45 ]	[ − 0.89; 0.98 ]	
HK	0.04	[ − 0.37; 0.46 ]	[ − 0.84; 0.93 ]	
HK2	0.04	[ − 0.46; 0.55 ]	[ − 0.89; 0.98 ]	
KR	0.04	[ − 1.27; 1.36 ]	[ − 40.77; 40.86 ]	
SJ	0.04	[ − 0.38; 0.47 ]	[ − 0.84; 0.93 ]	
SJ2	0.04	[ − 0.32; 0.41 ]	[ − 0.81; 0.90 ]	

Similarly, the prediction intervals show good agreement across all methods, with the exception of the KR method. The KR method generates a particularly erratic and uninformative prediction interval. This is caused by an unusually small value for the degrees of freedom *ν* = 1.54, which results in a hugely inflated value for $t_{\nu -1;\frac{\alpha }{2}}$. Also, observe that the HK prediction interval actually shrinks, but this is corrected when applying the HK2 method.

More importantly, based on the findings of the simulation study, given that the relative degree of heterogeneity is small, it is likely that (aside from the erratic prediction interval for the KR method) all the derived prediction intervals are too narrow and thus inaccurate (see the bottom right of Table 4). Therefore, one should be cautious about using prediction intervals in this setting and, at best, they should be viewed as approximate.

### 4.2 Post‐treatment meta‐analysis

**Figure 5:**
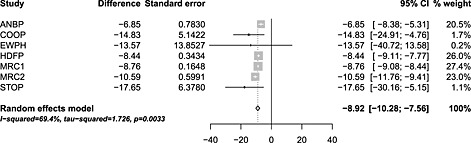
Forest plot for the post‐treatment difference in diastolic blood pressure between the treatment and control groups based on the random effects meta‐analysis model (1) fit using restricted maximum likelihood and confidence interval for the mean treatment effect calculated using the conventional method. N, conventional method; HK, Hartung–Knapp; HK2, modified Hartung–Knapp; KR, Kenward–Roger; SJ, Siddik–Jonkman; SJ2, modified Sidik–Jonkman.

**Figure 6:**
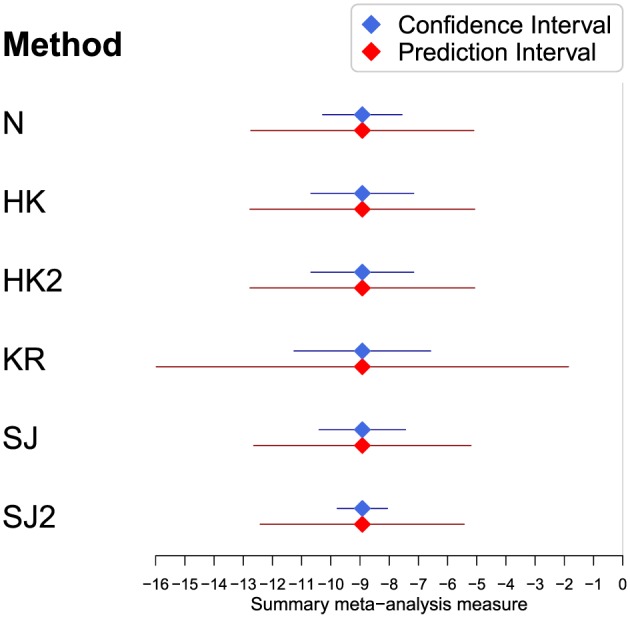
The summary results for the post‐treatment difference in diastolic blood pressure between the treatment and control groups based on a random effects meta‐analysis fit using restricted maximum likelihood with confidence and prediction intervals constructed using a variety of methods. N, conventional method; HK, Hartung–Knapp; HK2, modified Hartung–Knapp; KR, Kenward–Roger; SJ, Siddik–Jonkman; SJ2, modified Sidik–Jonkman.

The forest plot in Figure 5 shows the results of a meta‐analysis on the post‐treatment difference in DBP between the treatment and control groups, based on a random effects meta‐analysis fit using model (1) with REML. As expected, given the diversity of the populations, at the end of follow‐up, there is a large amount of between study heterogeneity in the treatment effect with *I* ^2^ = 69.4*%*[32.6*%*;86.1*%*] and $\hat{\tau }=1.73$. Table 7 and Figure 6 summarise the confidence and prediction intervals for each of the methods. All methods generate confidence intervals that are entirely below zero, providing strong evidence that the mean treatment effect is to reduce DBP more than control. However, note that the conventional method still produces a 95*%* confidence interval that is narrower than the others, and based on the simulation study, the other methods (especially HK) would appear more appropriate. The SJ2 method produces a much tighter confidence interval than the other methods; however, the simulation study results for heterogeneous studies of varied size show that the SJ2 method produces over‐precise results in this setting (see the bottom right of Table 3).

**Table 7:** The post‐treatment difference in diastolic blood pressure between the treatment and control groups $\left({\hat{\theta }}_{1}\right)$ along with the 95*%* confidence intervals (CI) and standard error (s.e.) for each study. The summary results $\hat{\theta }$ are based on a random effects meta‐analysis fit using restricted maximum likelihood with confidence and prediction intervals constructed using a variety of methods. Methods: N, Conventional method; HK, Hartung‐Knapp; HK2, Modified Hartung‐Knapp; KR, Kenward‐Roger; SJ, Siddik‐Jonkman; SJ2, Modified Sidik‐Jonkman.

Individual study results				
Trial	${\hat{\theta }}_{1}$	95% CI	s.e.	n
ANBP	− 6.85	[ − 8.38; − 5.31 ]	0.78	1530
COOP	− 14.83	[ − 24.91; − 4.76 ]	5.14	349
EWPH	− 13.57	[ − 40.72; 13.58 ]	13.85	172
HDFP	− 8.44	[ − 9.11; − 7.77 ]	0.34	4798
MRC1	− 8.76	[ − 9.08; − 8.44 ]	0.16	6991
MRC2	− 10.59	[ − 11.76; − 9.41 ]	0.60	2651
STOP	− 17.65	[ − 30.16; − 5.15 ]	6.38	268
Summary meta‐analysis results				
Method	$\hat{\theta }$	95 % CI	95% PI	
N	− 8.92	[ − 10.28; − 7.56 ]	[ − 12.74; − 5.10 ]	
HK	− 8.92	[ − 10.68; − 7.16 ]	[ − 12.77; − 5.07 ]	
HK2	− 8.92	[ − 10.68; − 7.16 ]	[ − 12.77; − 5.07 ]	
KR	− 8.92	[ − 11.26; − 6.58 ]	[ − 15.98; − 1.86 ]	
SJ	− 8.92	[ − 10.40; − 7.44 ]	[ − 12.64; − 5.20 ]	
SJ2	− 8.92	[ − 9.78; − 8.06 ]	[ − 12.42; − 5.43 ]	

Because heterogeneity is large in this meta‐analysis, a prediction interval may be a more appropriate summary than the confidence interval for the mean effect; in other words, it is of interest whether the treatment effect is likely to always be beneficial in new populations. Prediction intervals from all methods agree that treatment is likely to be effective in at least 95*%* of settings at reducing DBP in a new setting, as none contain zero. However, there is some concern that these prediction intervals may be too short because the simulation study reveals that in this setting, the coverage of prediction intervals is too low (for example, see the bottom right of Table 3). All methods produce reasonably similar prediction intervals, but they should clearly be viewed as approximate given the simulation study. Further, it is clear that the KR interval is wider than the others. Again, the simulation study identifies that the KR prediction interval is likely to be overly conservative in this setting.

## 5 Discussion

### 5.1 Key findings

**Figure 7:**
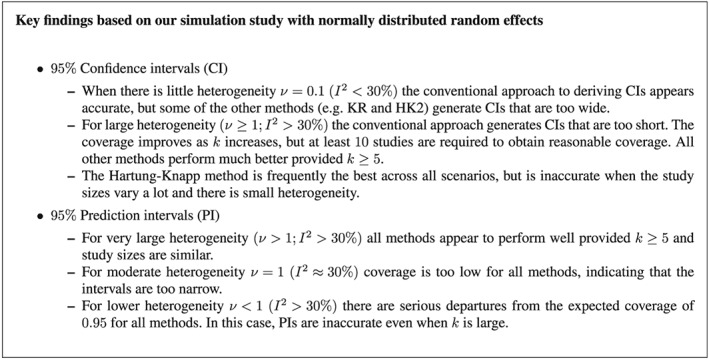
Key findings regarding the coverage performance of confidence intervals (CIs) and prediction intervals (PIs) obtained using the conventional and modified intervals for a random‐effects meta‐analysis model (1) with estimation via restricted maximum likelihood.

In this paper, we compared the performance of several different methods for constructing confidence intervals for the mean effect and prediction intervals for the effect in a new setting from a random effects meta‐analysis estimated using REML. In particular, we used a simulation study to examine the coverage performance of confidence and prediction intervals constructed using both conventional and novel estimators for the variance of the mean effect, combined with a normal or *t* distribution for deriving intervals. The key findings are summarised in Figure 7.

Firstly, it was demonstrated that when there is a large degree of heterogeneity (*ν* > 1;*I* ^2^ > 30*%* approximately for balanced studies), confidence intervals constructed by the conventional approach are consistently too short if there are few studies. The coverage improves as the number of studies increases, but at least 10 studies are required to obtain reasonable coverage. By contrast, other methods that correct the confidence interval to account for additional uncertainty perform reasonably well in terms of coverage, provided there are at least five studies. Of these methods, when the heterogeneity is large and when study sizes are similar, the standard HK method appears consistently the best in terms of coverage based on our simulation findings. This conclusion is in line with other, previous simulations studies 5, 6, 7. However, even the HK method is problematic in situations where heterogeneity is low, and the study sizes are quite varied, as the coverage appears slightly too low.

When there is very little heterogeneity, such as *ν* = 0.1(approximately 15*%* < *I* ^2^ < 18*%* for balanced studies), the conventional confidence intervals are generally very accurate in terms of coverage, even for just *k* = 3 studies. As a result, some of the methods that widen the conventional confidence interval (for example, KR and HK2) have coverage that is too large. However, the HK method remains consistently accurate in terms of coverage for this situation.

For prediction intervals, it was shown that all methods perform reasonably well in terms of coverage provided there are at least five balanced studies and a reasonably large degree of heterogeneity (*ν* > 1;*I* ^2^ > 30*%* approximately for balanced studies). However, the performance of all methods rapidly deteriorates as the between study variability is reduced and/or there is a large imbalance in the study sizes. For example, when *ν* = 1 prediction intervals are generally too short in all cases when k⩾5, even when there are a large number of studies. When the level of heterogeneity is even lower (*ν* < 1;*I* ^2^ < 30*%* approximately for balanced studies), prediction intervals are extremely inaccurate. Even when heterogeneity is large (*I* ^2^ = 60*%* for example), if there are a mixture of study sizes then the performance of the prediction intervals may still be poor, especially when there are fewer than 10 studies.

### 5.2 Recommendations

Ideally, for the reporting of a random effects meta‐analysis based on heterogeneous studies, both confidence and prediction intervals should be reported. Confidence intervals can be used to explain the uncertainty in the mean effect, while prediction intervals describe the potential effect in a new setting. However, the results of this paper identify that, in certain situations, one should interpret the confidence and/or prediction interval with caution.

When there is little heterogeneity, confidence intervals constructed using the conventional method are generally very accurate in terms of coverage. Indeed, some of the other methods (for example, KR and HK2) actually generate confidence intervals that are too wide in this case. Hence, provided there are sufficient studies with little variability in the treatment effect between them, the conventional method can be used to construct accurate confidence intervals.

On the other hand, when there is a moderate or high degree of heterogeneity, one should not routinely construct confidence intervals using the conventional method unless there are a large number of studies. Indeed, if heterogeneity is large and only a small number of studies are available then it would be prudent to construct confidence intervals by a number of different methods. The HK method appears generally very good in terms of coverage in this situation and indeed most other settings, unless there is a large imbalance in the study sizes.

If there is substantial between‐study heterogeneity then it is important to consider prediction intervals for the effect in a new population, in addition to the confidence interval. If the degree of heterogeneity is sufficiently large, one can construct accurate prediction intervals by almost any of the methods discussed here provided there are at least five studies that are reasonably well balanced in terms of size.

When the study sizes are unbalanced or there are lower levels of heterogeneity, all of the methods discussed here fail to construct accurate prediction intervals, including the conventional and increasingly used equation proposed by Higgins *et al.* 1 and Riley *et al.* 2. Even when heterogeneity is large, if the study sizes are quite different, then even the conventional method may still perform poorly, with under‐coverage again a concern. Therefore, in these settings, one should be cautious of interpreting frequentist prediction intervals, irrespective of how they are constructed; at best, they should be viewed as approximate, and further research is needed to address this in the frequentist setting.

For researchers who remain keen to produce prediction intervals, perhaps the most natural approach is to use a Bayesian method 16, 17, for example, with an empirically based prior distribution for the between‐study variance 18.

### 5.3 Limitations and further research

The simulation study in this paper considers the performance of the different models for a non‐specific normally distributed outcome measure. While this means that our findings and recommendations are applicable to a wide range of measures, it ignores issues associated with some, more problematic, outcome measures (e.g. rare binary outcomes). Moreover, for binary outcomes in general, the standard error will be strongly correlated with the effect estimate. The performance of the random effects models in such situations should be the subject of further, more‐tailored simulation studies.

Our simulation study also considered the impact of different mixtures of sample sizes on the model performance. These scenarios, which included incorporating one large or small study as well as a mixture of large and small studies, provide useful information for how these models might perform in practice. However, as our study did not modulate the within or between study variance to account for this, we were unable to control degree of heterogeneity in these cases.

Further, while we considered a broad range of values for *k*, the number of studies in the meta‐analysis, we did not consider every plausible value of *k*. However, although we did not consider a study size between 10 and 100, it is unlikely that the results would change dramatically on this range.

Another limitation of this study is that we only consider estimating *τ* ^2^ using REML. Additional simulations (not presented here) show that the results remain relatively consistent when estimating *τ* ^2^ using DerSimonian and Laird's method of moments 10. However, for a discussion on the impact of other heterogeneity estimators on the results of a random effects meta‐analysis,refer to Sánchez‐Meca and Marín‐Martínez 19 and Langan *et al.* 20.

Another important issue when conducting a random effects meta‐analysis is the specification of the random effects distribution. While it is common practice to assume the random effects are normally distributed, there is little justification for this 21. Consequently, there are other methods that relax the assumption of normal random effects, such as the Bayesian method proposed by Lee and Thompson 16. Thus, an appraisal of the impact of non‐normal random effects is of considerable interest for future research.

### 5.4 Conclusions

When assuming the conventional random effects model with normally distributed random effects, confidence intervals for the mean effect should account for the uncertainty in the estimate of between‐study variation. Generally, following REML estimation of a random effects meta‐analysis, we recommend the Hartung–Knapp method although further work is needed to address the slightly low coverage for this approach in the situation where there are a variety of study sizes or low between‐study heterogeneity. Further, prediction intervals derived using the conventional method proposed by Higgins *et al.* 1 are only accurate when the between‐study heterogeneity is relatively large and study sizes are similar. In other situations, the coverage of prediction intervals can be too low and so should be treated with caution, with derivation in a Bayesian framework potentially preferred.
